# Exploring the Health-Related Quality of Life of Patients Treated With Immune Checkpoint Inhibitors: Social Media Study

**DOI:** 10.2196/19694

**Published:** 2020-09-11

**Authors:** François-Emery Cotté, Paméla Voillot, Bryan Bennett, Bruno Falissard, Christophe Tzourio, Pierre Foulquié, Anne-Françoise Gaudin, Hervé Lemasson, Valentine Grumberg, Laura McDonald, Carole Faviez, Stéphane Schück

**Affiliations:** 1 Bristol-Myers Squibb Rueil-Malmaison France; 2 Kap Code Paris France; 3 Bristol-Myers Squibb Stockport United Kingdom; 4 Paris-Sud University Paris France; 5 Paris-Descartes Universitiy Paris France; 6 AP-HP Paris France; 7 INSERM U1178 Paris France; 8 Bordeaux University Bordeaux France; 9 Inserm U1219 Bordeaux France; 10 UFR Pharmacy, Grenoble Alpes University Grenoble France; 11 Bristol-Myers Squibb London United Kingdom

**Keywords:** health-related quality of life, immunotherapy, patients with cancer, social media use, measures, real world

## Abstract

**Background:**

Immune checkpoint inhibitors (ICIs) are increasingly used to treat several types of tumors. Impact of this emerging therapy on patients’ health-related quality of life (HRQoL) is usually collected in clinical trials through standard questionnaires. However, this might not fully reflect HRQoL of patients under real-world conditions. In parallel, users’ narratives from social media represent a potential new source of research concerning HRQoL.

**Objective:**

The aim of this study is to assess and compare coverage of ICI-treated patients’ HRQoL domains and subdomains in standard questionnaires from clinical trials and in real-world setting from social media posts.

**Methods:**

A retrospective study was carried out by collecting social media posts in French language written by internet users mentioning their experiences with ICIs between January 2011 and August 2018. Automatic and manual extractions were implemented to create a corpus where domains and subdomains of HRQoL were classified. These annotations were compared with domains covered by 2 standard HRQoL questionnaires, the EORTC QLQ-C30 and the FACT-G.

**Results:**

We identified 150 users who described their own experience with ICI (89/150, 59.3%) or that of their relative (61/150, 40.7%), with 137 users (91.3%) reporting at least one HRQoL domain in their social media posts. A total of 8 domains and 42 subdomains of HRQoL were identified: Global health (1 subdomain; 115 patients), Symptoms (13; 76), Emotional state (10; 49), Role (7; 22), Physical activity (4; 13), Professional situation (3; 9), Cognitive state (2; 2), and Social state (2; 2). The QLQ-C30 showed a wider global coverage of social media HRQoL subdomains than the FACT-G, 45% (19/42) and 29% (12/42), respectively. For both QLQ-C30 and FACT-G questionnaires, coverage rates were particularly suboptimal for Symptoms (68/123, 55.3% and 72/123, 58.5%, respectively), Emotional state (7/49, 14% and 24/49, 49%, respectively), and Role (17/22, 77% and 15/22, 68%, respectively).

**Conclusions:**

Many patients with cancer are using social media to share their experiences with immunotherapy. Collecting and analyzing their spontaneous narratives are helpful to capture and understand their HRQoL in real-world setting. New measures of HRQoL are needed to provide more in-depth evaluation of Symptoms, Emotional state, and Role among patients with cancer treated with immunotherapy.

## Introduction

Health-related quality of life (HRQoL) is a complex subjective concept pertaining to multiple domains including physical, emotional, social, professional, and functional well-being [1,2]. The number of cancer cases is continuing to rise across Europe with approximately 8% of people currently living with cancer [3] and with the side effects of cancer treatment (eg, hair loss, pain, fatigue, nausea) [4]. Patients and their families also often experience psychological distress as a result of a cancer diagnosis and its treatment, including stress, anxiety, and depression [5]. Therefore, HRQoL is particularly important to understand and to be quantified among people living with cancer or caring for someone with cancer. Several cancer-related HRQoL self-administered questionnaires, such as the European Organization for Research and Treatment of Cancer Quality of Life Questionnaire Core 30 (EORTC QLQ-C30) [6] and the Functional Assessment of Cancer Therapy - General (FACT-G) [7], have been used and validated in oncology patient populations, but are limited in that they are required to be complete at a predefined time.

Recently, the internet has provided an opportunity for introducing new sources of clinically relevant information related to patients’ perception of illness and its burden [8]. Specifically, patients and their relatives are increasingly using forums, blogs, and social media to obtain health-related information and support [9]. Indeed, the information generated online represents an alternative way to understand patients’ and relatives’ health state compared with self-administered questionnaires. These patient-generated health data are produced spontaneously—thus not limited to medical consultations, for instance—mostly anonymously, and may better correspond to patients’ and relatives’ feelings than close-ended questions. Moreover, text mining techniques applied to analyze social media data can be used with relative ease [10] and have opened up new opportunities to bridge the gap between qualitative and quantitative data [11].

In parallel to the development of new strategies for collecting information on HRQoL of patients with cancer, treatment with immunotherapy has emerged as an innovative curative approach that, instead of destroying cancer cells directly, stimulates the immune system making it capable of identifying and selectively attacking cancer cells. The body develops an internal defense mechanism which can lead to reduced side effects and improved HRQoL [12]. Recent studies have assessed HRQoL of patients undergoing immunotherapy using standard questionnaires, but exclusively only in clinical trial settings [13-17]. In addition to prolonged survival, HRQoL results showed that immune checkpoint inhibitors (ICIs), such as nivolumab, maintained or improved baseline HRQoL levels in patients with advanced melanoma [18], advanced renal cell carcinoma [19], or advanced squamous non-small-cell lung cancer [20]. However, HRQoL of patients treated with ICI remains largely unknown under real-world conditions and it is not known whether the EORTC QLQ-C30 and the FACT-G questionnaires capture the full range of HRQoL domains and experiences relevant to these specific patients. The use of social media data offers a novel opportunity to explore this.

This study assessed and compared conceptual coverage of ICI-treated patients’ HRQoL between standard questionnaires and users’ ICI-related experiences described in social media. We hypothesized that, given the evolving and dynamic nature of the HRQoL concept, new HRQoL subdomains would emerge from social media posts going beyond the coverage of existing questionnaires, especially in a population of patients treated with new drugs such as ICI.

## Methods

### Study Design and Population

This was a retrospective study using a text mining approach to retrieve information from social media posts written by French language internet users between January 1, 2011, and August 31, 2018. The start date of January 2011 was selected because it corresponds to the date of marketing authorization for selling the first available immuno-oncology treatment in France. Included posts (comprising forum posts or comments on videos or photos) had to mention past or current patients’ experience with ICI (ie, ipilimumab, nivolumab, pembrolizumab, atezolizumab, or those available through early access schemes or clinical trials, such as durvalumab, tremelimumab, and avelumab). Posts referring to treatments other than ICI were excluded. Posts could be authored by patients themselves or by their relatives, here interchangeably referred to as *patients*. Posts were retrieved from the following social media: 12 generic French medical web forums; 4 cancer-specialized French medical web forums; and 3 generic social media (Facebook, YouTube, and Instagram). These social media were screened from the Detec’t database [21]. Description of forums is provided in Multimedia Appendix 1. All posts had to be publicly available and include at least one of the predetermined keywords with their synonyms (see the list in Multimedia Appendix 2). Ambiguous posts or duplicates (ie, similar posts posted by the same user in different social media) were excluded through a manual review.

### Data Extraction

**Figure 1:**
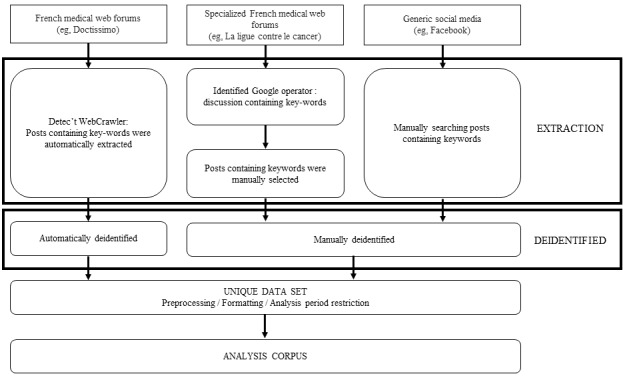
Diagram of the steps for creating the analysis corpus.

Automatic and manual extraction methods were utilized, depending on the source of the posts. For generic French medical web forums, posts were automatically extracted using the Detec’t WebCrawler [22]. Collection of the posts was performed according to the HTML structure of each forum. All posts containing one of the predefined keywords were automatically retrieved from discussion threads and deidentified (signature and quote withdrawal). The deidentification of posts was performed by using an in-house algorithm based on regular expression to automatically identify specific sequences of characters (proper names, phone numbers, postal codes, mail addresses, etc). For specialized French medical web forums, posts containing predefined keywords were identified using a Google operator searching these keywords in selected forum websites (Site: +URL). Identified discussion threads were manually explored, and posts containing one of the predefined keywords were manually retrieved and deidentified. Finally, for the 3 generic social media, posts were identified and retrieved by manually searching the predefined keywords using the social media embedded search fields. These posts were also manually deidentified. Data from these 3 collection methods were then grouped in a unique data set (the analysis corpus), which went through several steps of cleaning (preprocessing by removing French accents, unnecessary spaces, and punctuation and lowercasing all words; removal of stop words; and stemming, based on Porter’s algorithm [23]) and formatting (transformation of the corpus into a matrix; creation of tokens and measure of their frequency; exclusion of hardly used, ambiguous, and misspelled words; and document-term matrix weighting; Figure 1).

### Study Variables

The analysis corpus contained information on data source (name of the forum or social media), post characteristics (URL of the page or discussion where the post was published; date of the publication; pseudonym or alias of the user; keyword associated with the post leading to its extraction), patient characteristics (age, gender, type of cancer), and user status among “patient,” “relative,” and “unspecified.” Regarding content of retrieved posts, for each patient “associated HRQoL domains” and “associated subdomains” were collected manually. “Associated HRQoL domains” were grouped considering the classification of domains provided in existing measures (ie, the EORTC QLQ-C30 and the FACT-G). For each domain, “associated subdomains” mentioned by patients were also collected even if not included in standard measures in order to allow new subdomains to emerge.

### Comparison With HRQoL Standard Questionnaires

The EORTC QLQ-C30 [6] and the FACT-G [7] are among the most widely used questionnaires to capture HRQoL of patients with cancer in research and clinical settings [24,25]. Compared with other questionnaires, they are not limited to a specific cancer type [26,27] and cover the highest number of HRQoL domains across all cancer-specific questionnaires [28]. Both questionnaires were first developed in 1993 [29] and are validated in the French language [30,31].

The EORTC QLQ-C30 is a 30-item questionnaire composed of multi-item scales and single items, and comprises 5 functional scales (physical activity, role, emotional state, cognitive state, and social state), 3 symptoms scales (fatigue, nausea and vomiting, and pain), and a global health status and HRQoL scale. The remaining single items assess additional symptoms commonly reported by patients with cancer: dyspnea, insomnia, lack of appetite, constipation, diarrhea, and financial difficulties. The FACT-G is a 27-item questionnaire divided into 4 well-being subscales: physical well-being, social/family well-being, emotional well-being, and functional well-being [32]. Coverage of domains identified in social media and related subdomains measured by the EORTC QLQ-C30 and the FACT-G was manually assessed independently by 2 operators and compared through a concept mapping approach [33].

### Data Analysis

Each post corresponded to a statistical unit. Frequentist analysis was performed on extracted posts to characterize the whole analysis corpus through the following indicators: number of posts, number of patients, occurrence of HRQoL domain(s), keywords (including the number of extracted posts), data source, and users’ characteristics. A Venn diagram was generated through the CRAN package “VennDiagram.” The list of identified subdomains, including the number of patients and posts, was presented in a descriptive format using Microsoft Excel. For each subdomain, coverage by one or several items of the 2 questionnaires was assessed. For each domain, coverage rates were calculated by dividing the sum of subdomain occurrences covered by questionnaires by the total number of occurrences in the social media. The diagram indicating the coverage rates of each HRQoL domain retrieved from social media posts through the standard questionnaires EORTC QLQ-C30 and FACT-G was generated through Microsoft Excel.

## Results

### Description of the Population and Posts

The final analysis corpus included 267 social media posts meeting the inclusion criteria, with a maximum of 11 posts from 1 patient and a median of 2 posts per patient. Through the manual extraction, we identified 150 patients (posters) who described their personal experience with ICI (89/150, 59.3%) or that of their relative (61/150, 40.7%). A majority of patients were women (82/150, 54.7%) and gender was undetermined for only 8/150 patients (5.3%). The type of related cancer was identified for 123/150 patients (82.0%): the most frequent cancers were lung cancer and melanoma (Table 1). The majority of posts were retrieved from 1 cancer-specific patient forum (La ligue contre le cancer, 78 posts by 43 patients) and 1 generic medical forum (Doctissimo, 76 posts by 43 patients). The most frequently identified keyword was “immunotherapy” (72/150, 48.0%) followed by “nivolumab” (31/150, 20.7%) and “ipilimumab” (20/150, 13.3%).

**Table 1:** Patients characteristics.

Characteristics			Patients mentioning their quality of life (N=150)
**Gender, n (%)**			
	Women	82 (54.7)	
	Men	60 (40.0)	
	Undetermined	8 (5.3)	
**Type of cancer, n (%)**			
	Lung cancer	46 (30.7)	
	Melanoma	41 (27.3)	
	Others	36 (24.0)	
	Undetermined	27 (18.0)	
**Type of immunotherapy, n (%)**			
	Nivolumab	31 (20.7)	
	Ipilimumab	20 (13.3)	
	Pembrolizumab	16 (10.7)	
	Others	11 (7.3)	
	Undetermined	72 (48.0)	
**Posts number, n**			267
	Per patient, median (min-max)	2 (1-11)	
**Social media, n (%)**			
	Doctissimo	43 (28.7)	
	La ligue contre le cancer	43 (28.7)	
	Facebook	16 (10.7)	
	Others	48 (32.0)	

### Domains and Subdomains of HRQoL

**Figure 2:**
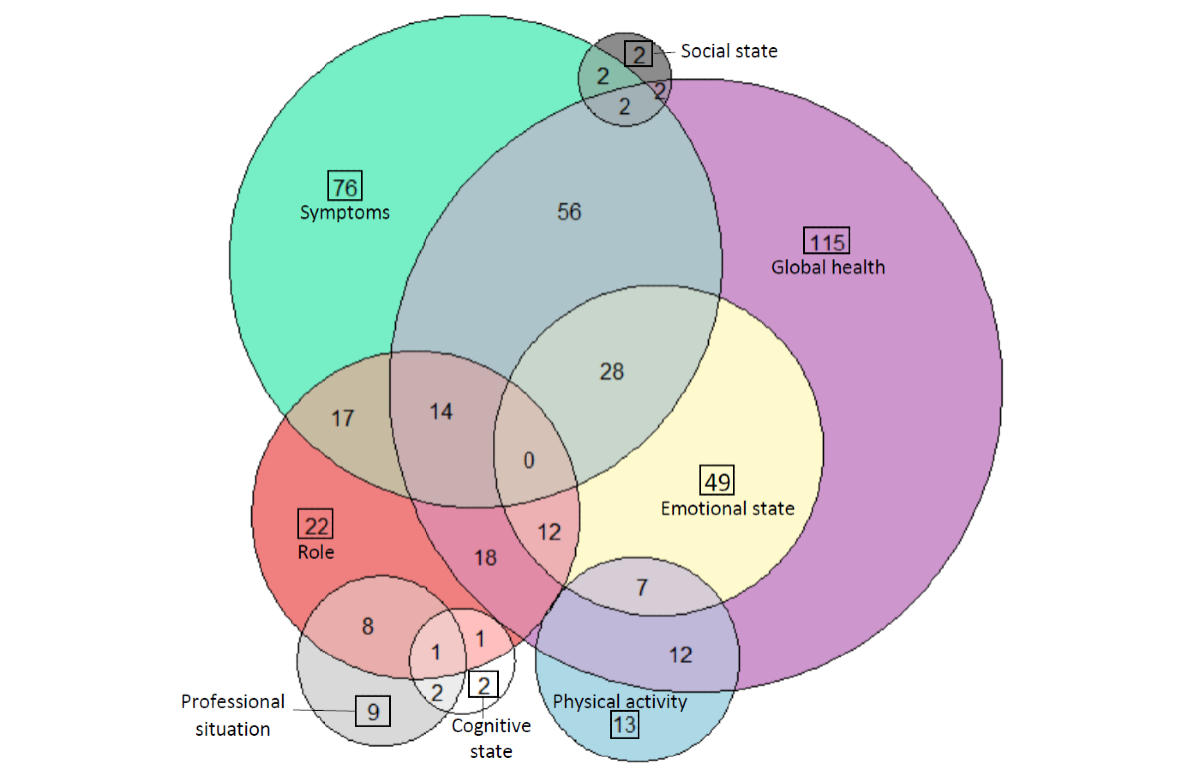
Venn diagram showing the distribution of patients according to their posts mentioning HRQoL domains. HRQoL: health-related quality of life.

Of the study population, 91.3% (137/150) mentioned at least one HRQoL domain in their posts. We identified 8 HRQoL domains, with 83.9% (115/137) patients mentioning Global health, 55.5% (76/137) mentioning Symptoms, 35.8% (49/137) mentioning Emotional state, 16.1% (22/137) mentioning Role, 9.5% (13/137) mentioning Physical activity, 6.6% (9/137) mentioning Professional situation, 1.5% (2/137) mentioning Social state, and 1.5% (2/137) mentioning Cognitive state. A Venn diagram illustrates the distribution of the HRQoL domains and the relationship among them (Figure 2). Verbatim examples for each HRQoL domains are presented in Table 2.

A total of 42 subdomains of HRQoL were mentioned in the social media posts and were distributed as follows: 1 for Global health, 13 for Symptoms, 4 for Physical activity, 10 for Emotional state, 7 for Role, 3 for Professional situation, 2 for Cognitive state, and 2 for Social state. Table 2 provides the list and occurrences of each social media subdomain. Among 115 patients mentioning their Global health, a majority (63/115, 54.8%) considered it to be poor, while 27.8% (32/115) and 17.4% (20/115) perceived it to be stable and good, respectively. As for Symptoms, 76 patients mentioned symptoms 123 times, which were grouped into 13 subdomains. The most addressed subdomain of Symptoms was Fatigue/Tiredness (34/123, 27.6%). A total of 49 patients mentioned at least one subdomain concerning Emotional state. With 15 occurrences, Hope was the most recurrent subdomain (15/49, 31%). The most frequent subdomains of Role were normal life/reduced activities and pace of life (12/22, 55%). Only 13 patients mentioned Physical activity and the most reported subdomains pertaining to this larger domain were minimal or no physical activity/maintained activity (5/13, 38%) and difficulty walking/eating (5/13, 38%). Professional situation was mentioned by 9 patients with the most frequent subdomain being sick leave (6/9, 67%). Two subdomains were mentioned for the Cognitive state and the Social state, respectively.

Table 2 also synthetizes coverage of each social media subdomain by items of the QLQ-C30 and the FACT-G questionnaires. The QLQ-C30 showed a wider coverage of social media subdomains than the FACT-G (45% [19/42] versus 29% [12/42], respectively).

**Table 2:** Verbatim examples of posts related to HRQoL^a^ of patients with cancer.

HRQoL domain and specific subdomain			Verbatim example (translation from French)	
**Global health**				
	Deteriorated	“Hello, we started immunotherapy 3 weeks ago, we didn’t combine it with [Drug], his health is deteriorating more and more. Yesterday he was re-hospitalized...I don’t think his condition could get any worse...I strongly hope he gets better, that he can eat and move...”		
**Symptoms**				
	Weight loss	“When I was diagnosed with melanoma in May 2010, the [hospital] immediately referred me to the [protocol] ([Drug] treatment). (...) it must be said that I lost 9 kilos after the 1^st^ injection, then 2 kilos per month, until we stopped this treatment, in [month] 2011, for a total weight loss of 28 kilos. Since the beginning of the injections I had to stop working.”		
**Emotional state**				
	Optimism	“I have lung cancer and metastasis (...). I have had immuno with [drug] for 69 weeks, I am quite well and the mass sometimes decreases slightly and sometimes I have almost no nodule on the lungs. I have almost no reactions as I do not lose my hair, no nausea, just a little bit. I am at stage 4, but I trust my treatment and I have a lot of hope.”		
	Exhaustion	“I have been fighting lung cancer with bone metastases for more than three years. Since the beginning of July, I have been trying immunotherapy. If people have had this treatment, I would like to know what the side effects were. As for me, I am very tired, have no appetite and my moral is deteriorating.”		
**Physical activity**				
	Minimal or no physical activity/maintained activity	“Cancer was diagnosed in [month] 2017. An operation was not possible. In [month] after the immunotherapy the saturation level was very low and the oxygen allowed maintaining a very minimal physical activity... a few steps...”		
**Role**				
	Normal life	“I have been receiving this treatment for 1 year and a half, I don't know if it will cure me but thanks to [Drug] I have been in remission for more than a year. Relatively non-toxic, it allows to lead a normal life in parallel.”		
	Reduced activities and pace of life	“Hello. I have melanoma stage 4; for 3 years, interferon, [Drug], [Drug] currently every 15 days. I am tired, so exhausted that I can no longer take care of my house, but I have the rage to live.”		
**Professional situation**				
	Maintained work activity	“In short, of course I'm afraid of dying, but that's out of the question. This type of cancer is treatable if taken in time. And I was in stage 4 Clark. Immunotherapy is not always cool, but I still work, I manage the house, the children and I am still smiling.”		
**Cognitive state**				
	Concentration disorders	“Here I am, dean of this post: a year under [drug]. And no trace of melanoma! (...) If I had to relive it I would stop working earlier because I have the impression that [drug] has mostly prevented me from recovering, from sleeping well, with a cumulative effect on the end where I ended the year on the kneecaps wondering if I was not depressed so much I could not concentrate on anything.”		
**Social state**				
	No longer participates in family parties	“On the first scan, increased lung metastases...but no new ones, so that's something. (...) We won’t do much for the holidays. Until recently we used to go to my in-laws, there were a lot of us, but since my mother-in-law died and since I'm no longer in Olympic shape, we haven’t been moving around!”		

^a^HRQoL: health-related quality of life.

### Coverage of HRQoL Domains in Social Media by the QLQ-C30 and FACT-G Questionnaires

As shown in Table 3, Global health was entirely covered by both the QLQ-C30 and the FACT-G. Physical activity, Professional situation, Cognitive state, and Social state were also fully covered by the QLQ-C30. For Symptoms, the EORTC QLQ-C30 covered a majority (68/123, 55.3%) of the subdomains identified in social media posts, and so did the FACT-G (72/123, 58.5%). Coverage was lower for the Emotional state domain: 14% (7/49) by the EORTC QLQ-C30 and 49% (24/49) by the FACT-G. Finally, the EORTC QLQ-C30 covered 77% (17/22) of subdomains for Role, whereas the FACT-G covered 68% (15/22). Of these domains, the FACT-G fully covered only the Professional situation, whereas it covered 46% (6/13) of the Physical activity, with no coverage of neither Cognitive state nor Social state.

**Table 3:** HRQoL^a^ subdomains in social media posts and their coverage by EORTC QLQ-C30^b^ and FACT-G^c^ questionnaires.

HRQoL domains and subdomains in social media (number of occurrences in posts)		EORTC QLQ-C30 coverage (question number)		FACT-G coverage (question number)	
**Global health**					
	Deteriorated/stable/better (115)		✓ (Q29)		✓ (GF7)
**Symptoms**					
	Fatigue/Tiredness (34)		✓ (Q10, Q12, Q18)		✓ (GP1)
	None/Unspecified adverse events (23)		X		✓ (GP5)
	Fever (8)		X		X
	Pain/Joint pain/Leg pain/Liver pain (15)		✓ (Q9, Q19)		✓ (GP4)
	Cough (7)		X		X
	Loss of appetite/Weight loss (11)		✓ (Q13)		X
	Rash/itch (6)		X		X
	Respiratory trouble/Breathlessness (6)		✓ (Q18)		X
	Headache (3)		X		X
	Thyroid disorders (4)		X		X
	Heavy legs (2)		X		X
	Hair loss (2)		X		X
	Diarrhea (2)		✓ (Q17)		X
**Emotional state**					
	Optimism/Hope (15)		X		✓ (GE3)
	Exhaustion (7)		X		X
	Distress (6)		X		X
	Good mental health/Morale (5)		X		✓ (GE1, GE2)
	Fear (4)		✓ (Q22)		✓ (GE5, GE6)
	Depression (3)		✓ (Q24)		X
	Psychological disorders (3)		X		X
	Stable health (3)		X		X
	Isolate oneself (2)		X		X
	Emotional exhaustion due to side effects (1)		X		X
**Role**					
	Normal life/Reduced activities and pace of life (12)		✓ (Q6, Q7)		✓ (GF6, GF3)
	Time constraints regarding medical care (3)		X		X
	Financial problems (2)		✓ (Q28)		X
	Maintained activities (2)		✓ (Q6, Q7)		✓ (GF6)
	Home care constraints (1)		X		X
	Improved HRQoL (1)		✓ (Q30)		✓ (GF7)
	Ability to drive again (1)		X		X
**Physical activity**					
	Minimal or no physical activity/maintained activity (6)		✓ (Q1, Q2, Q3, Q4)		✓ (GP1, GP7)
	Difficulty walking/eating (5)		✓ (Q2, Q3, Q5)		X
	Difficulty climbing stairs (1)		✓ (Q2, Q3, Q5)		X
	Difficulty getting into bed alone (1)		✓ (Q2, Q3, Q5)		X
**Professional situation**					
	Sick leave (6)		✓ (Q6)		✓ (GF1, GF2)
	Maintained work activity (2)		✓ (Q6)		✓ (GF1, GF2)
	Reduction in working time (1)		✓ (Q6)		✓ (GF1, GF2)
**Cognitive state**					
	Concentration problems (1)		✓ (Q20)		X
	Memory problems (1)		✓ (Q25)		X
**Social state**					
	Requiring family assistance for medical care (1)		✓ (Q26)		X
	Inability to participate in family celebrations (1)		✓ (Q26)		X

^a^HRQoL: health-related quality of life.

^b^EORTC QLQ-C39: European Organization for Research and Treatment of Cancer QLQ-C30.

^c^FACT-G: Functional Assessment of Cancer Therapy - General.

**Figure 3:**
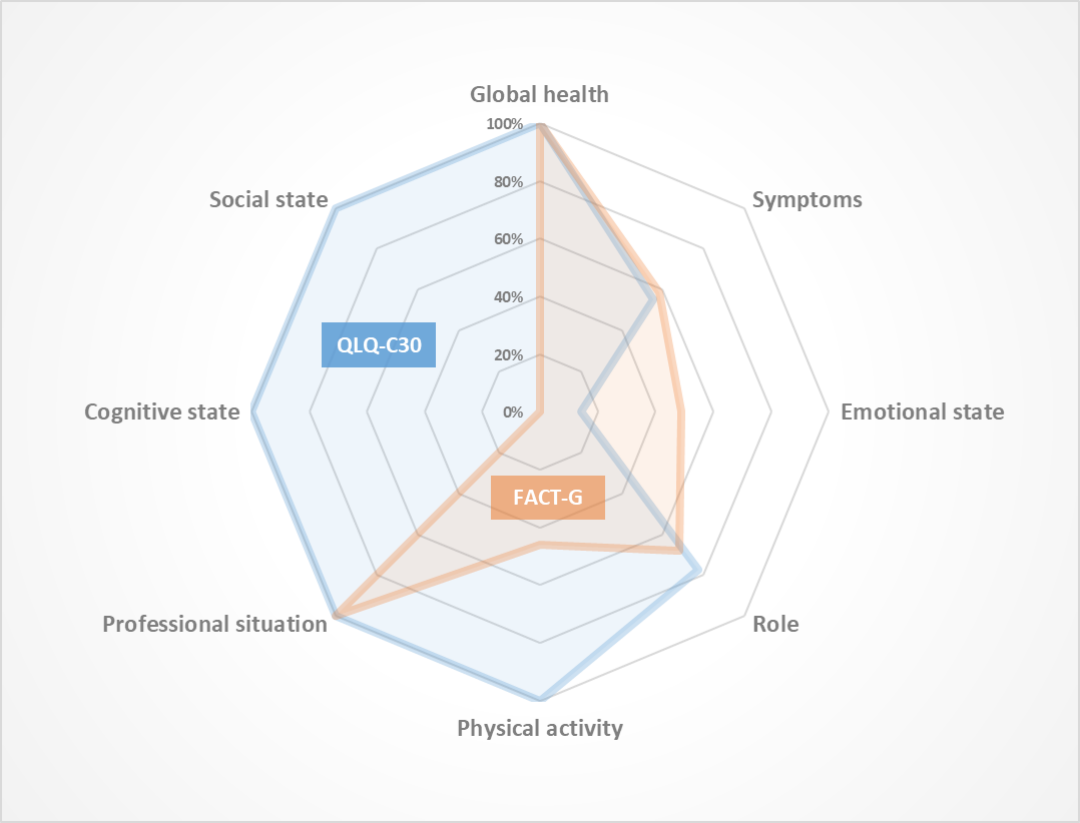
Diagram indicating the coverage rates of each HRQoL domains retrieved in social media by the standard questionnaires EORTC QLQ-C30 and FACT-G. FACT-G: Functional Assessment of Cancer Therapy - General; QLQ-C30: Quality of Life Questionnaire Core 30.

Specific subdomains which were not covered by both the EORTC QLQ-C30 and the FACT-G were fever, cough, rash/itch, headache, thyroid disorders, heavy legs, and hair loss for the Symptoms domain; exhaustion, distress, psychological disorders, stable health, emotional exhaustion due to side effects, and isolation for the Emotional state domain; and time constraints regarding medical care and ability to drive again for the Role domain (Figure 3).

## Discussion

### Principal Findings

This novel approach of using social media identified that some of the content posted by patients with cancer and caregivers with experience of ICI overlaps with concepts captured in the 2 most frequently used HRQoL questionnaires. However, the main findings also included the fact that there are a large number of concepts which are not captured in these 2 HRQoL questionnaires. These results confirmed our hypothesis by underlining the emergence of new subdomains of HRQoL in patients treated with immunotherapy. In particular, we observed that retrieved social media posts frequently addressed specific subdomains of the HRQoL domains of Symptoms, Emotional state, and Role, which are not fully covered by the EORTC QLQ-C30 and the FACT-G. Reasons for not including these subdomains in standard questionnaires are varied. First, questionnaires such as the EORTC QLQ-C30 and the FACT-G are designed to be short, effective, and time-saving, thus reducing burden on patients completing these questionnaires. As a consequence, they are limited and do not cover the whole range of the issues impacting patients’ HRQoL. Second, these questionnaires were developed more than 25 years ago, which may explain why the domains might not fully capture the impact of recently developed therapies, such as ICI. The concept of HRQoL is evolving and several users have already raised the problem of “partial covering” [33], which relates to the complexity of measuring this broad ranging concept through a robust methodology [34]. Third, subdomains retrieved by our study might be specific to immunotherapy and, therefore, not measured by generic standard questionnaires. Finally, accurately quantifying an individual’s HRQoL is, per se, a debated question, because standardized questionnaires might restrict a patient’s choice and limit their spontaneity, thus not using a patient-centered approach [35].

We observed a remarkable usage in the occurrences of the keyword “immunotherapy” which likely results from the growing availability of ICI, the steadily increased use of ICI in the French health care system, and, in parallel, the growing proportion of social media users.

### Comparison With the Literature on HRQoL and Immunotherapy

The conceptual and psychometric measurement properties of the EORTC QLQ-C30 and the FACT-G have not yet been systematically examined in ICI-treated patient populations and the results of this study cast some doubt on the content validity of these measures in ICI-treated patients. Indeed, content validity is the extent to which an instrument measures the important aspects of concepts most significant and relevant to a patient’s condition and its treatment [36]. Because there are subdomains which do not appear in the 2 questionnaires that participants completed, we can assume that the 2 questionnaires lack content validity for this specific patient population. This does underline the need for new or adapted patient-reported outcomes in patients treated with immunotherapy.

Existing studies using these questionnaires in patients with cancer treated with immunotherapy are still often limited to research settings [17,18,37]. For instance, the work by Long and colleagues [18] has demonstrated that the use of nivolumab maintained baseline HRQoL levels to provide long-term quality of survival benefit among 418 patients with advanced melanoma. Cella and colleagues [19] have confirmed the association between nivolumab treatment and HRQoL improvement using the FACT-G among 847 patients with advanced renal cell carcinoma.

Although HRQoL has already been assessed with standard questionnaires in a number of clinical trials of immunotherapy, covered domains were pre-established and limited. Instead, in our study various new spontaneous subdomains emerged, such as fever, time constraints of treatment, difficulty in driving, and isolation. Furthermore, certain subdomains covered by the questionnaires might be inadequately designed for patients treated with ICI. For example, in the FACT-G, hope is collected in a negative way (ie, “I am losing hope in the fight against my illness”), whereas social media users mainly referred to hope in an optimistic way (ie, “regaining hope”).

### Limitations of the Study

This study was not without limitations. First, selection bias was a major limitation because analyses were restricted to selected data sources and available contents. The population under study was composed of social media users who might not necessarily reflect the characteristics of all patients with cancer receiving immunotherapy. However, because social media are increasingly used by patients [38], especially in France [39], retrieved posts should pertain to an important section of the French population. We also collected data from relatives who provided immunotherapy-related experiences of patients with cancer who were not active on social media. However, given the small number of patients and relatives in our sample, we were not able to distinguish their posts within the analysis corpus. Our results should then be interpreted considering this further limitation. HRQoL self-assessed by patients might be different from the evaluation provided by relatives, as shown in previous research [40]. Similarly, the limited size of our analysis corpus did not allow an analysis per type of cancer. Melanoma, for instance, has long been treated with immunotherapy [41], which means that HRQoL of patients with melanoma might be different from HRQoL related to other cancers.

Second, an extraction bias is also possible because we only considered posts containing predefining keywords. If users expressed their experiences with immunotherapy and consequent impact on HRQoL by using other nonspecific words, their posts were not included in the final analysis corpus. To mitigate this bias, the set of keywords was as comprehensive as possible.

Third, because this study was based on secondary use of data published in social media, it was impossible to get additional data and information from patients (only identified by their pseudonym). The analysis was then restricted to what users mentioned, which can lead to missing data or incomplete capture of the patients’ full experience. In particular, subdomains emerging from social media were spontaneously addressed by users versus items from standard questionnaires. The fact that some items of the EORTC QLQ-C30 or the FACT-G were not mentioned in social media posts does not mean that patients were not concerned by them. For this, our conclusions should be considered with a certain degree of caution.

Fourth, because immunotherapy is a fairly recent therapeutic approach, few posts could be identified. A larger analysis corpus is needed to obtain more robust results and to validate our initial findings. As demonstrated in this study, the number of posts concerning immunotherapy is increasing year after year and new studies will benefit from this expanding analysis corpus. In particular, posts should be compared across countries where the EORTC QLQ-C30 and the FACT-G are usually administered to capture HRQoL. Exploration of potential cross-country differences in subdomains mentioned in social media would be noteworthy.

Fifth, social media represent an ideal place where patients can freely and spontaneously discuss their experiences with their therapy, thus providing valuable information on their HRQoL. However, this observation should be interpreted cautiously, because social media data may include a higher frequency of erroneous information, and patients posting on social media forums may not be representative of the wider patient population [10].

Finally, biases related to semantic analyses must be considered. Given the low number of posts within our analysis corpus, we were obliged to retrieve and code the mentions manually and could not apply automated analysis, for example, using topic modeling.

### Implications and Future Research

We were able to include users’ subjective narratives in the evaluation of the impact of ICI on patients’ HRQoL. The results of our study suggest that commonly used measures such as the EORTC QLQ-C30 and the FACT-G may require updating to improve their coverage and applicability of HRQoL domains under real-world conditions. The challenge in measuring HRQoL lies in its uniqueness to individuals [35] and questionnaires such as the EORTC QLQ-C30 and the FACT-G might not take account of this by imposing standardized models of HRQoL. For this reason, as already demonstrated in studies concerning other diseases than cancer [42-46], posts in online forums and social media should be integrated in the assessment of patients’ HRQoL, because they can help either detect adverse events or characterize patient experience in a more individualized and spontaneous way.

In summary, this study suggested to explore further specific HRQoL domains related to patients treated with ICI to potentially enrich existing standard questionnaires with new items that are more relevant for these patients in their daily confrontation with disease and treatment.

### Conclusion

Patients with cancer and their relatives are using social media to share their experiences with immunotherapy and its impact on HRQoL, particularly with regard to Global health and Symptoms. Emotional state and Role are also increasingly referenced in online forums and social media. Collecting and analyzing these spontaneous narratives can be helpful to capture how immunotherapy affects patients’ HRQoL in a more individualized way, thus obtaining information on more facets of life that are important for patients. While standard questionnaires can provide objective scores, which are easily interpretable from a clinical and research point of view, mining social media posts might better inform health care professions and patients of the impact of immunotherapy on patients’ HRQoL under real-world conditions. Future research is required to corroborate our findings and propose new individualized measures covering HRQoL more in depth than existing standard questionnaires.

## Appendix

Multimedia Appendix 1 Description of the websites used to retrieve the posts under study.

Multimedia Appendix 2 List of predefined keywords for posts inclusion.

## Abbreviations

EORTC: European Organization for Research and Treatment of Cancer
FACT-G: Functional Assessment of Cancer Therapy - General
HRQoL: health-related quality of life
ICI: immune checkpoint inhibitors
QLQ-C30: Quality of Life Questionnaire Core 30
