# Supplementary material for: Exploring the Health-Related Quality of Life of Patients Treated With Immune Checkpoint Inhibitors: Social Media Study
Source: J Med Internet Res. 2020 Sep 11;22(9):e19694. doi: 10.2196/19694 (PMC7519426; doi:10.2196/19694)
Supplement: Multimedia Appendix 1 [file jmir_v22i9e19694_app1.docx]

| Name | Type | Description | Posts posted (n) | Annual active users (n) |
| --- | --- | --- | --- | --- |
| Allo docteurs | Generalist |  |  |  |
| Atoute | Generalist | Website created in 2000 by a medical doctor with health-related articles and forum. | 5,744,559 | 82,183 |
| Doctissimo | Generalist | Health information and exchange web site created in 2000. | 313,315,020 | 2,907,48 |
| Patientsworld | Generalist | The first French platform of services dedicated to health. Formerly Entrepatients, from 2013 it has become Patientsworld. | Not public | 1 million |
| Espoirs | Generalist | Self-help forum available from 2013. | 37,547 | 414 |
| France Lymphome espoir | Cancer-specialized | Association of patients with lymphoma aiming at assisting and informing. | 81,690 | 4,412 |
| Futura Sciences | Generalist | Web information portal dedicated to knowledge structured in five sections including health. | 6,382,118 | 900,635 |
| Journal des femmes | Generalist | French women's website founded in 2003 with a health-related section. | Not public | Not public |
| La Ligue contre le cancer | Cancer-specialized | French association created in 1918 for promoting cancer screening and for supporting patients. | Not public | Not public |
| Les impatientes | Cancer-specialized | Network for women with breast cancer. | Not public | 10,750 |
| Notre temps | Generalist | Online version.of a French magazine for senior audiences. | Not public | Not public |
| Onmeda | Generalist | Health and well-being website launched in 2015. | Not public | 24,290 |
| Point vert | Generalist | Forum on pharmacy in general | 7 852 | 721 |
| Psychoactif | Generalist | Self-support organization created in 2006 aiming at discussing about drug consumption. | 521,430 | 26,428 |
| Santé médecine | Generalist | Website created in 2003 for health exchanges for patients and healthcare professionals. | 426,000 | Not public |
| Vaincrelemelanome | Cancer-specialized | Association of patients with melanoma aiming at assisting and informing. | Not public | Not public |
