# Supplementary material for: Exploring the Health-Related Quality of Life of Patients Treated With Immune Checkpoint Inhibitors: Social Media Study
Source: J Med Internet Res. 2020 Sep 11;22(9):e19694. doi: 10.2196/19694 (PMC7519426; doi:10.2196/19694)
Supplement: Multimedia Appendix 2 [file jmir_v22i9e19694_app2.docx]

| Key-word | Synonyms |
| --- | --- |
| Immuno oncologie | Immuno-onco*, immuno onco*, Immunoonco* |
| Anti PD1 | Anti-PD1, AntiPD1, Anti PD 1 |
| Anti PDL1 | Anti-PDL1, Anti PD L1, AntiPDL1, Anti PDL 1 |
| Anti CTLA4 | Anti-CTL*, antiCTL* |
| Checkpoint inhibiteur | Checkpoint inhib*, Checkpoint inib*, checpoint inhib* |
| Yervoy | Yerv* |
| Ipilimumab | Ipi* |
| Opdivo | Opd*, Obdivo |
| Nivolumab | Nivo* |
| Keytruda | Ketr*, Keitr* |
| Pembrolizumab | Pembro* |
| Tecentriq | Tecentr* |
| Atezolizumab | Atezo* |
| Imfinzi | Imfi* |
| Durvalumab | Durva*, Duvra* |
| Bavencio | Baven* |
| Avelumab | Ave* |
| Tremelimumab | Tremel* |
